# Preoperative lymphocyte-to-monocyte ratio versus platelet-to-lymphocyte ratio as a prognostic predictor for non-small cell lung cancer patients

**DOI:** 10.2478/jomb-2019-0031

**Published:** 2020-01-23

**Authors:** Haixi Yan, Linling Cai, Shuaishuai Chen, Jun Li

**Affiliations:** 1 Taizhou Enze Medical Center(Group), Taizhou Hospital of Zhejiang Province, Department of Clinical Laboratory, Linhai, Zhejiang Province, China

**Keywords:** lymphocyte/monocyte, platelet count/lymphocyte, non-small cell lung cancer, prognosis overall survival - disease-free survival, prognoza: opšta stopa preživljavanja - preživljavanje bez bolesti, nemikrocelularni karcinom pluća, broj trombocita/limfocita, limfociti/monociti

## Abstract

**Background:**

We investigated the prognostic value of the preoperative lymphocyte-to-mononuclear ratio (LMR) and platelet-to-lymphocyte ratio (PLR) in a large cohort of patients with non-small cell lung cancer (NSCLC).

**Methods:**

Clinical-pathological data from 507 NSCLC patients at Taizhou Hospital of Zhejiang Province between 2010 and 2016 were retrospectively evaluated. X-tile software was used to assess the optimal cutoff levels for LMR and PLR. Univariate and multivariate Cox regression models were used to assess the prognostic factors.

**Results:**

The median follow-up duration after surgical resection was 34.5 months. Patients were stratified into 2 groups by LMR (2.6 and > 2.6) and PLR (179.6 and > 179.6). Our results revealed that lower LMR (HR = 3.163 (1.821-5.493), P = 0.000), age (HR = 2.252 (1.412-3.592), P = 0.001), T stage (HR = 3.749 (2.275-6.179), P = 0.000), N stage (HR = 3.106 (1.967-4.902), P = 0.000), and cut edge (HR = 3.830 (1.077-13.618), P = 0.038) were considered to be independent indicators for overall survival (OS) of NSCLC patients. For disease-free survival (DFS), age, sex, T stage, N stage, LMR and cut edge were verified to be independent prognostic factors in patients with NSCLC.

**Conclusions:**

In the study cohort, reduced LMR was a robust independent predictor for both OS and DFS in patients with NSCLC who underwent surgical resection.

## Abstract (sr)

**Uvod:**

Ispitali smo prognostičku vrednost preoperativnog odnosa limfocita i mononuklearnih jedinjenja (LMR) i odnosa trombocita i limfocita (PLR) u velikoj grupi pacijenata sa nemikrocelularnim karcinomom pluća (NSCLC).

**Metode:**

Izvršena je retrospektivna evaluacija 507 NSCLC pacijenata u Taizhou bolnici u provinciji Zhejiang između 2010. i 2016. godine. Korišćen je X-tile softver za procenu optimalnih graničnih nivoa za LMR i PLR. Za procenu prognostičkih faktora korišćeni su univarijatni i multivarijatni Cox regresioni modeli.

**Rezultati:**

Srednje vreme praćenja nakon hirurške resekcije bilo je 34,5 meseci. Pacijenti su stratifikovani u 2 grupe u zavisnosti od LMR (2,6 i > 2,6) i PLR (179,6 i > 179,6). Naši rezultati su pokazali da su niži LMR (HR = 3,163 (1,821-5,493), P = 0,000), starost (HR = 2,252 (1,412-3,592), P = 0,001), T stadijum (HR = 3,749 (2,275-6.179), P = 0,000), N faza (HR = 3,106 (1,967-4,902), P = 0,000), i granične vrednost (HR = 3,830 (1,077-13,618), P = 0,038) smatrani nezavisnim pokazateljima ukupne stope preživljavanja (OS) pacijenata sa NSCLC. Za preživljavanje bez bolesti (DFS), starost, pol, T stadijum, N faza, LMR i cut-off vrednost su potvrđeni kao nezavisni prognostički faktori kod pacijenata sa NSCLC.

**Zaključak:**

U kohorti studije redukovani LMR je bio robustan nezavisni prediktor i za OS i za DFS kod pacijenata sa NSCLC koji su bili podvrgnuti hirurškoj resekciji.

## Introduction

Lung cancer is a malignant tumour that originates in the bronchial mucosa or gland. Common symptoms of lung cancer are cough, dyspnoea, weight loss and chest pain. Symptomatic patients often have the chronic obstructive pulmonary disease (COPD) [1]. Lung cancer is the leading cause of cancer death worldwide, with a 5-year survival rate of only 18% [2]. In China, the results of the Third National Cause of Death Sampling Survey in 2008 showed that the incidence of lung cancer increased 26.9% each year, and lung cancer is the deadliest cancer [3]. Based on the biological characteristics, treatments and prognosis of lung cancer, the WHO divides lung cancer into non-small cell lung cancer (NSCLC) (approximately 85%) and small cell lung cancer. The survival rates of lung cancer are poor overall, but prognosis varies strongly by disease stage at diagnosis. In general, patients with stage I and stage II disease are most likely to be cured by surgery [4]. However, a substantial number of patients with NSCLC will experience recurrence or metastasis after surgery. Therefore, potentially useful biomarkers are sought to predict the prognosis of patients with NSCLC after surgery.

Recent reports show that tumour characteristics and the host inflammatory response determine the progression and prognosis of cancer, particularly in NSCLC patients with a background of chronic inflammation [5] [6] [7]. Inflammatory biomarkers, such as lymphocyte-to-mononuclear ratio (LMR) and platelet-tolymphocyte ratio (PLR), are prognostic factors in colorectal liver-only metastases, breast cancer, thymic epithelial tumours, biliary tract cancer, and ovarian cancer [8] [9] [10] [11] [12]. Therefore, convenient and simple inflammatory biomarkers associated with risk stratification are important for the prognosis of tumours. In this study, we discussed the prognostic value of the preoperative LMR and PLR in patients with NSCLC after surgery.

## Materials and Methods

### Subjects

A retrospective analysis was conducted in patients with newly diagnosed lung cancer at Taizhou Hospital between January 2010 and March 2017. The inclusion criteria were as follows: 1) patients were aged 18 years or older; 2) NSCLC was diagnosed by histopathology, including adenocarcinoma, large cell carcinoma and squamous cell carcinoma (SCC); 3) no infections of autoimmune diseases were diagnosed; 4) no radiotherapy or chemotherapy was performed before surgery; 5) radical resection was the surgical method; 6) complete laboratory data were examined before surgery; and 7) patients agreed to follow-up. A total of 507 patients with lung cancer were enrolled: 309 males and 198 females aged 28-83 years.

All procedures that followed were in accordance with the ethical standards of the responsible committee on human experimentation (institutional and national) and with the Helsinki Declaration of 1964 and later versions. Informed consent or a suitable substitute was obtained from all patients for inclusion in the study.

### Treatment and follow-up

Each patient was followed up regularly until May 2015 or until death according to the National Comprehensive Cancer Network (NCCN) guidelines. Physical examination, radiologic examination and serum tumour biomarker examination, including examination for carcinoembryonic antigen (CEA) and SCC, were performed every 3 to 6 months for the first 3 years, every 6 months for the fifth year, and then annually. Clearly defined overall survival (OS) and disease-free survival (DFS) were used to represent the prognosis of patients with NSCLC. For patients who were alive at the end of follow-up, OS was defined as the time from the first diagnosis until the final followup. For patients who have died by the end of the follow-up, OS was defined as the time from the first diagnosis until death from any cause. When a patient had a recurrence (whether death or survival), DFS was defined as the time from first diagnosis to the recurrence. When a patient who died was without recurrence, DFS was defined as the time from the first diagnosis to the death. When a patient neither died nor there was recurrence DFS was defined as the time from the first diagnosis to the last follow-up.

### Statistical analysis

SPSS 22.0 was used for statistical analysis. The optimal cut-off values for PLR and LMR were calculated by X-tile 3.6.1 software [13] (Yale University, New Haven, CT, USA). Continuous variables are presented as the median with a range of 25-75%. The relationship between clinical-pathological parameters (features) and these inflammatory biomarkers was determined by the X^2^ test. To assess relative risk, 95% confidence intervals (95% CI) of hazard risk (HR) were used as common measures. Significant prognostic predictors associated with OS and DFS were included to perform multivariate analyses using the Cox proportional hazards regression model. P < 0.05 was considered statistically significant.

## Results

### Determination of the cut-off value

OS: The X-tile program was used to determine the optimal cut-off values for PLR and LMR of OS, which were 179.6 and 2.6, respectively. Patients were divided into two groups for further analysis (LMR 2.6 and > 2.6; PLR 179.6 and > 179.6).

DFS: The X-tile program was used to determine critical values for LMR and PLR, which were 5.2 and 184, respectively.

### Patient and tumour characteristics

Among the 507 patients, 309 (60.9%) were males, and 198 (30.1%) were females. The median age was 62 (IQR 55-69). The median follow-up of OS was 34.5 months (23.5-47.5 months), with 211 recurrences and 98 cancer deaths. The median follow-up of DFS was 31.0 months (19-42 months). According to the 7^th^ American Joint Committee on Cancer (AJCC) tumour classification, there were 283 (55.8%) and 224 (44.2%) T1 and T2-T4 stage distributions of NSCLC cases, respectively, and 412 (81.3%) and 95 (18.7%) N0 and N1-N2 stage distributions, respectively. A total of 407 (80.3%) patients underwent lobectomy, 89 (17.5%) patients underwent sub-lobar resection, and 11 (2.2%) patients underwent pneumonectomy. The majority of patients (88.2%) were confirmed not to have a venous invasion, and 68 (11.8%) were confirmed to have a venous invasion. There were 176 patients who underwent chemotherapy and 34 patients who underwent radiotherapy.

### Baseline patient characteristics according to LMR and PLR

The association of the LMR and PLR with clinical-pathological characteristics is listed in Table 1. Our results showed that LMR was significantly associated with sex, smoking history, alcohol history, histology and N stage. PLR was associated with histology and N stage (P < 0.05).

**Table 1:** Comparison of baseline clinical-pathological characteristics based on LMR and PLR

Factor	LMR		P	PLR		P
	≤2.6 >2.6			≤179.6 >179.6		
Age (years)			0.468			0.525
≤60	19	194		184	29	
>60	32	262		248	46	
Sex			0.000			0.741
Male	44	265		262	47	
Female	7	191		170	28	
Smoking history			0.000			0.101
Current	14	260		240	34	
No	37	196		192	41	
Alcohol			0.017			0.223
Yes	32	352		331	53	
No	19	101		98	22	
Histology			0.000			0.014
Adeno	20	338		314	44	
Other	31	118		118	31	
T stage			0.104			0.063
T1	23	260		250	33	
T2.T3.T4	28	196		182	42	
N stage			0.039			0.026
N0	36	376		358	54	
N1.N2	15	80		74	21	

### Prognostic value of LMR and PLR

Univariate analysis of clinic-pathological parameters using the Cox regression model was used to further predict OS and DFS. In univariate analysis, age, sex, smoking history, histology, T stage, N stage, PLR, LMR, vascular invasion, neural invasion, pleural inva-sion and cut edge were significantly associated with OS (P < 0.05), while age, sex, smoking history, histology, T stage, N stage, LMR, vascular invasion, neural invasion and cut edge were associated with DFS (P < 0.05). A multivariate Cox regression model was used to investigate clinical-pathological parameters. Our results revealed that low LMR (HR = 3.163 (1.821-5.493), P = 0.000) was associated with redu ced OS. Age (HR = 2.252 (1.412-3.592), P = 0.001), T stage (HR = 3.749 (2.275-6.179), P = 0.000), N stage (HR = 3.106 (1.967-4.902), P = 0.000), and cut edge (HR = 3.830 (1.077-13.618), P = 0.038) were considered to be independent indicators for OS in renal cell carcinoma (RCC) patients. For DFS, age, sex, T stage, N stage, LMR and cut edge were verified to be independent prognostic factors in patients with NSCLC.

## Discussion

In the development of the molecular biology of tumours, inflammatory responses play a key role in tumour occurrence and progression. We aimed to study the effect of the inflammatory markers LMR and PLR on the survival of patients with NSCLC. This study shows that preoperatively reduced LMR was an independent risk factor for poor clinical outcome in NSCLC patients undergoing surgical resection, but PLR was not significantly related to NSCLC patients.

In different studies, the cut-off values of LMR and PLR were distinct. Guthrie et al. [14] reported different studies using different cut-off values (2.0-5.0). For every cohort of patients, there is no global way to set up a satisfactory general threshold. In 507 patients who underwent NSCLC surgery, we chose X-tile software to determine the best cut-off value for the inflammatory markers of LMR and PLR. Many studies did not include time as a factor when selecting cut-off values, for example, receiver operating characteristics (ROC) curve. The X-tile program chose an exact cutoff in this study. We demonstrated that LMR was correlated with OS or DFS in patients with NSCLC.

In recent decades, the relationship between in ammation and cancer has been hypothesized. Several studies have proven the relationship between cancer and inflammation. In our study, we found that pre-treatment LMR was significantly associated with OS and DFS in patients with NSCLC. Other clinicpathological factors, such as the T stage, N stage, and positive surgical margins, were also significantly associated with OS and DFS in patients with NSCLC.

Lymphocytes play critical roles in the antitumour immunity of the host by infiltration into the tumour microenvironment after being triggered by immunologic antitumour reactions.

Previous studies have elucidated that lymphocytes are key factors in immunosurveillance and that the occurrence of an immunologic antitumour reaction depends on lymphocytic infiltration into the tumour microenvironment [15] [16].

However, systemic inflammation will depress cellular immunity, resulting in lymphocytopenia, which was marked as a decrease in CD4^+^ helper lymphocytes and an increase in CD8^+^ suppressor lymphocytes [17]. In contrast, tumour-associated macrophages (TAMs) are circulating monocytes that are recruited to tumour sites in excess [18] [19]. As a result, elevated circulating serum levels of monocytes may re ect increased production of tissue TAMs and high tumour burden and lead to worse survival outcomes. Therefore, LMR and PLR can reflect the body's antitumour status and affect the prognosis of patients with lung cancer.

Several limitations of the present study should be solved. First, this data set includes only surgically resected patients, so this cohort cannot represent patients who have unresectable lung cancer or patients who have not undergone lung cancer surgery. Second, this study includes only patients undergoing radical resection of lung cancer in Taizhou Hospital, with geographical limitations. Third, some of the fam-ilies of patients who died after follow-up refused to follow up on the phone, resulting in the patient not being included in the study, which led to some bias.

In conclusion, the findings in our study suggest that pre-treatment LMR is an independent prognostic biomarker for OS and DFS in NSCLC patients. However, further work is needed to illuminate a more detailed relationship between in ammatory biomarkers and prognosis in NSCLC patients.

## Ethical approval

All procedures performed in studies involving human participants were in accordance with the ethical standards of the institutional and/or national research committee and with the 1964 Helsinki declaration and its later amendments or comparable ethical standards.

## Conflict of interest statement

The authors stated that they have no conflicts of interest regarding the publication of this article.
